# Impact of Ginger Root Powder Dietary Supplement on Productive Performance, Egg Quality, Antioxidant Status and Blood Parameters in Laying Japanese Quails

**DOI:** 10.3390/ijerph18062995

**Published:** 2021-03-15

**Authors:** Zabihollah Nemati, Zahra Moradi, Kazem Alirezalu, Maghsoud Besharati, António Raposo

**Affiliations:** 1 Department of Animal Science, Ahar Faculty of Agriculture and Natural Resources, University of Tabriz, Tabriz 5166616471, Iran; zahra.moradi81@yahoo.com (Z.M.); m_besharati@hotmail.com (M.B.); 2 Department of Food Science and Technology, Ahar Faculty of Agriculture and Natural Resources, University of Tabriz, Tabriz 5166616471, Iran; kazem.alirezalu@tabrizu.ac.ir; 3 CBIOS (Research Center for Biosciences and Health Technologies), Universidade Lusófona de Humanidades e Tecnologias, Campo Grande 376, 1749-024 Lisboa, Portugal

**Keywords:** antioxidant, egg quality traits, ginger, immunity, Japanese quail performance

## Abstract

Medicinal plants with antibacterial effects have been used by humans for centuries. In the recent decade, due to the development of antibiotic resistant strains, many studies have focused on the use of natural compounds as feed additives in livestock. Ginger, among all, have repetitively shown numerous biological activities, antibacterial, and antibiotic properties. This study was conducted to evaluate the effects of ginger root powder (GP) on the performance, egg quality, and blood parameters of Japanese quail. A total of 240 10-weeks old female quails were used in a completely randomized design with 4 treatments, 4 replicates, and 15 birds per replicate. Dietary treatment were basal diet (control) and basal diet containing 0.5, 1, and 1.5 g/kg of ginger root powder. Growth performance and exterior and interior quality of egg were measured biweekly over eight-week period. At the end of experiment blood parameters were evaluated. The results showed that diet supplementation with different levels of GP had no significant effect on egg production, egg mass weight, and egg weight (*p* > 0.05). However, feed intake and feed conversion ratio were significantly lower in the treatment group than the control in the whole period (*p* < 0.05). Egg Quality traits (shape index, albumen index, the percentage of albumen, yolk and shell, yolk pH, and shell thickness and strength) were not affected by the supplements in the whole trial period. Addition of GP significantly increased the albumen height, Haugh unit, and albumen pH in comparison with the control treatment (*p* < 0.05). GP reduced blood triglyceride level yet was ineffective on blood total antioxidant capacity and malondialdehyde. In conclusion, dietary supplementation with GP, could improve productive performance and the egg quality of Japanese quails. Nonetheless a comprehensive study needs to be performed in order to evaluate the impact of quail dietary ginger supplementation on productive performance and egg quality and their stability during storage time for commercial use.

## 1. Introduction

Today, many phytochemical rich medicinal herbs are considered as potential alternatives to antibiotics and growth promoters due to the ban of antibiotics use in livestock in the European union [1]. Aromatic plants have been successfully used to improve antioxidant capacity in poultry industry [2,3]. Ginger *(zingiber officinale roscoe)* belongs to the Zingiberaceae family, including 47 genera and 1400 species [4]. Ginger is a native tropical plant in Southeast Asia and its commercial cultivation is not limited to Asia as is grown worldwide [4]. The global production of ginger was 3.3 million tons in 2016 [5]. The ginger can be consumed as fresh produce, dehydrated, and or processed product. Fresh rhizome is widely used as a spice and food condiment whether in the form of powder, extract, supplement, and or medicine [6]. Not only that the ginger rhizome is nutrient rich (amino acids, fatty acids, vitamins, and minerals) but the produce also contains compounds such as gingerol, gingerdiol, gingerdione, and shagaol that are potent intestinal mucous membrane and digestion stimulators [7]. The gingerols and shagaols are responsible for the pungency of fresh ginger and dried ginger, respectively [8].Ginger containing different phenolic component and they exhibited many bioactivities such as antioxidant [9], anti-anxiety [10], anti- Nausea [11], anti-inflammation [12], glucose-lowering [13], and health benefiting effects by reducing free radicals damage and improving cardiovascular status [14,15]. Furthermore, ginger is used for its various medicinal properties and alleviation and treatment of different symptoms such as animal mycotoxicosis [16], vomiting, pain, indigestion, and upper respiratory tract infection [17].

Throughout the production of ginger drink, powder, flakes and or ginger extract, significant amount of process discards, and byproducts are produced every year that could potentially be used as inexpensive feed additive in animal industry [18,19]. In a recent study, ginger supplement in broiler chicken diets stimulated the immune and digestive systems of the birds considerably [20]. In another study, Yusuf et al. [21] showed that, ginger in combination with probiotic and organic acid (citric acid) in laying Japanese quail diets improved the laying performances, feed conversion ratio (FCR), egg quality, bone characteristics, and reproductive indexes. Accordingly, diet supplementation with ginger powder increased total superoxide dismutase and glutathione peroxidase (Gpx) activities while it reduced malondialdehyde (MDA) and cholesterol concentrations in serum of broiler chickens at 21 and 42 days of age [22]. Ginger has also been reported to increase gastrointestinal secretion, digestive enzymes, improve circulation and intestinal movements [23]. It has been reported that using 4 and 8 g/kg of ginger powder of diet reduced feed intake but improved weight gain compared with control group [24]. In a study, ginger powder at 0.05 g/kg of diet, increased the egg production, hatching, reproductive performance, and economic efficiency of Japanese quail [25]. Other studies have reported that the addition of ginger essential oils at 300 µL/kg of diet, increased egg shell weight and shell thickness in laying hens [26]. Supplementation of broiler chickens diet with ginger reduced MDA and cholesterol concentration in serum of chickens at 21 and 42 days of age [22]. The effects of ginger supplementation in diets of broiler chicks at 64 weeks of age at 0.2, 0.4, and 0.6% showed a significant improvement in the feed conversion ratio in the treatment group containing 0.4% ginger powder [25]. Ibtisham et al. [27] have shown improvement of the production rate and feed intake of ginger powder and Chinese herbal medicine fed laying hens. They also concluded that ginger powder could be a suitable alternative to the antibiotic in poultry feed. In another study, Habibi et al. [28] reported that ginger powder and essential oils may be a vital replacement for synthetic antioxidants in broiler diets. Additionally, these researchers stated that ginger powder might be better than extracted essential oil for improving antioxidant status in broiler. The effect of diet supplementation with extract from thyme and ginger on the egg quality of laying hen was investigated by Damaziak et al. [29], who demonstrated that hen diet supplementation was improved Haugh unit of albumen and yolk color of fresh and hard boiled eggs. Recent studies suggest that Haugh unit and yolk color are important in the commercial production of table eggs which affected by dietary composition (e.g., protein sources and pigments) [30]. Color is an important qualitative feature of food acceptability as it affects consumers’ perceptions of quality and flavor helping those making decisions about the purchase. Most consumers find yolk color related to the age and health of the animal and the quality of egg and its derivatives. Generally, edible additives and feeding methods are the main factors in the egg yolk color. For instance, natural pigments of plants such as carotenoids play an important role in the development of egg yolk color [25]. However, to the best of our knowledge, information regarding the potential benefits of these feed additive in the Japanese quail’s diet is limited, so the present study was conducted to evaluate the effects of using different levels of ginger powder on the productive performance, eggs quality, and blood parameters in laying Japanese quails.

## 2. Materials and Methods

Animal welfare statement: All the methods and protocols used in this study were approved by the Research Bioethics Committee of Tabriz University (RBCT) for Use of Laboratory Animals of University of Tabriz (Approved number: IR.TABRIZU.REC.1399.032).

### 2.1. Experimental Birds and Management

A total of 240 laying Japanese quails (10 weeks old), after a two-week adaptation period, were randomly divided into 4 experimental groups (4 replicates each, 15 birds per pen). Dietary groups included the basal diet (control) and the basal diet supplemented with 0.5, 1, and 1.5 g ginger root powder per kg of diet. The birds were kept in a multi-story wired cages in a well-ventilated room with the temperature of 23–29 °C and 16L:8D light regime during experiment. Each cage had 3600 cm^2^ floor space with size 45 × 45 cm^2^ and was equipped with two nipple drinker and one feeder. The basal diet was formulated to meet the National Research Council (NRC) recommendations [31] as showed in Table 1. Quails received water and feed ad libitum throughout the experimental period.

### 2.2. Preparation of Ginger Powder Samples

Fresh ginger root was purchased from the local market and then cut into slices. Ginger slices were air-dried (sunshade for 1 day) at room temperature and further oven-dried (at 40 °C for 40 h) (Memmert UNB400). Dehydrated slices were then processed by grinder device (IKA, MF 10 basic) to fine powder, and subsequently stored in moisture-controlled ziplock bags at 4 °C. Ethanolic ginger extract was prepared by mixing 2 g of ginger powder in the 100 mL of absolute ethanol. The mixture was processed for 30 min at 85 °C. The prepared extract was filtered by Whatman No. 1 filter paper. Dry extract (5 g) was dissolved in 10 mL of methanol solvent and sonicated for 30 min at 15 °C. Constituents of the extract were quantified by HPLC (Smartline, Knauer, Germany) method [32]. The contents of bioactive components were determined in the extract and the concentration of 6-gingerol, 8-gingerol, 10-gingerol, and 6-shogaol was 6163.1, 802.8, 783.9, and 839.9 mg/kg ginger (on dry weight basis), respectively. Nutrient compositions and total phenolic content of ginger root powder were analyzed according to standard method, Association of Official Analytical Chemists (AOAC) [33] and Nemati et al. [34], respectively and the results are given in Table 2.

### 2.3. Measurement of Productive Performance and Egg Quality

In this study, egg production was recorded daily; however, feed consumption and egg weight [25] were recorded weekly. Moreover, feed efficiency was calculated by dividing the total feed intake by total egg mass during each period. The feed conversion ratio (FCR) [35] was expressed as kilograms of feed consumed per kilogram of egg produced. Six eggs (per replicate) were randomly selected to determine the traits related to the characteristics of quail eggs at 0, 2, 4, 6, and 8 weeks. Egg length and width were measured with a 0.01 mm digital caliper (Mitutoyo). For Haugh unit measurements six eggs were weighed and then cracked separately. Albumen heights of three near yolk areas were measured with a 0.01 mm digital caliper. The Haugh unit [35] was calculated using egg weight and albumen height data of each 6 egg. Furthermore, the yolk height and width were measured by 0.01 mm digital caliper. For calculation of yolk to albumen ratio as well as yolk and albumen percentages, yolk and albumen were carefully separated from each other and subsequently weighed by 0.001 g digital scale (A&D N92) [36]. For color measurements using Roche color fan, eggs were individually broken onto a flat surface and then color was recorded [35]. Egg shell thickness was measured using a 0.01 mm micrometer at three different points. Average of three points was considered as final thickness of each eggshell. The eggshell percentages were calculated using washed and dried eggshell weights [35]. For pH analysis, yolk and albumen were completely mixed with a glass rod prior to measurement separately (pH meter; Hanna 211, Woonsocket, RI, USA) [34].

### 2.4. Blood Biochemical Parameters Analysis

At the end of the experiment, two birds from each replicate were randomly picked up for slaughter (6 birds per treatment). Blood samples were collected from the neck vein of the birds using sterilized needles and then centrifuged for 15 min at 3000 rpm to separate the serum. The collected serum was stored at −20 °C for the analysis of glucose (Catalog No: 117500), albumin (Catalog No: 5017) and total protein (Catalog No: 128500), cholesterol (Catalog No: 110500), and triglycerides (Catalog No: 132500). Blood biochemical parameters were measured by a spectrophotometric analysis, using commercially available kits (Pars Azmun Diagnostic, Tehran, Iran).

### 2.5. Antioxidant Status

Ferric reducing antioxidant power assay was used to assess the total antioxidant capacity (TAC) of blood samples [37]. TAC was quantified by the reaction of phenanthroline and Fe^2+^ using a spectrophotometer at 520 nm. TAC is defined as the amount of antioxidants required to increase the absorbance by 0.01, in 1 mL of blood sample at 37 °C. Concentration of MDA in the serum, as an index of lipid peroxidation and oxidative stress, was determined using the thiobarbituric acid reactive substances (TBARS) method [37]. The principle is that TBARS reacts with MDA to form a stable pink color that could be measured spectrophotometrically at 532 nm. The values of MDA were expressed as (nmol/mL).

### 2.6. Statistical Analysis

Data of production performance and egg quality were subjected to one-way analysis of variance (ANOVA) using MIXED procedure (Repeated Measurement), SAS (version 9) as a completely randomized design [38]. Tukey multiple comparison test was used to compare the averages at a 5% confidence level. The Shapiro–Wilk and Levene tests were used for model assumptions of homogeneity of variance and normality, respectively. The percentage data including egg mass, egg production rate, yolk index, egg yolk, eggshell and albumen relative weight, and egg shape were transformed by arcsine of the square root before analysis to achieve homogeneity of variance. No statistical difference was observed between the two sets of data thus results of statistical analysis on the original data are presented in this article.

The statistical design model is as follows:

Y_ijkm_ = μ + T_i_ + W_j_ + TW_ij_ + Øk_(ji)_ + E_ijkm_.

In which Y_ijkm_ is observed parameters, µ is the mean of population, T_i_ is the treatment effect, W_j_ is the time effect, TW_ij_ is the treatment and time interactions, Øk_(ji)_ is the random factor (bird), and E_ijkm_ are residual effects.

Data of blood biochemical parameters and immune response were subjected to one-way ANOVA (general linear model (GLM) procedure), SAS following the statistical model. Comparison of means was performed using Duncan test at the confidence level of 5%. A level of *p* < 0.05 was used as the criterion for statistical significance.

Y_ij_ = µ + T_i_ + E_ij_.

In which Y_ijk_ is observed parameters (dependent variable), µ is the mean of population, T_i_ is the treatment effect and E_ij_ are residual effects.

## 3. Results

### 3.1. Productive Performance

The effect of dietary supplementation with natural additives of ginger powder on the productive performance is presented in Table 3. The results showed that the experimental treatments had no significant effect on egg production rate among the treatments during the experimental period (*p* > 0.05). Egg mass and egg weight were not significantly affected by the level of ginger powder used (*p* > 0.05). As shown in Table 3, addition of different levels of ginger significantly reduced feed intake during the whole experimental period (*p* < 0.05) compared with the control treatment. Ginger powder had dose-dependent effect since highest concentration of 1.5 g/kg of diet was most the potent treatment. The effect of treatment and time interaction was insignificant on the feed intake, egg weight and egg mass (*p* > 0.05). Nonetheless, the treatment x time interaction was significant on the FCR (*p* < 0.05). According to our results, Table 3, GP supplemented diet reduced FCR compared with the control group during experiment period (*p* < 0.05). High levels of ginger powder at 1.5 g/kg level decreased FCR compared with other groups. The experimental treatments significantly reduced FCR during breeding weeks. Moreover, the effect of time on the FCR was also significant.

### 3.2. Quality Traits of Eggs

The effects of diet supplementation with different levels of ginger powder on qualitative traits of eggs are given in Table 4. The results showed that the effects of ginger powder on egg weight, shape index, albumen weight, shell weight, albumen, yolk and shell percentage, yolk pH, and shell thickness and strength were insignificant (*p* > 0.05). Ginger supplementation improved yolk index; however, it did not increase yolk weight compared with the control treatment (*p* > 0.05). Nonetheless, effect of time on yolk index was significant (*p* < 0.05). The highest yolk color was related to the highest level of ginger in the diet. The effect of treatment x time interaction, as shown in Table 5, were significant on yolk color, albumen height and albumen index during experimental period (*p* < 0.05). The albumen and yolk pH were significantly reduced by ginger powder experimental diet compared with the control treatment (*p* < 0.05). Ginger diet improved Haugh unit relative to control group (*p* < 0.05), but egg specific gravity (ESG) did not differ (Table 4).

### 3.3. Blood Parameters and Egg Yolk Phenolic Compounds

**Figure 1:**
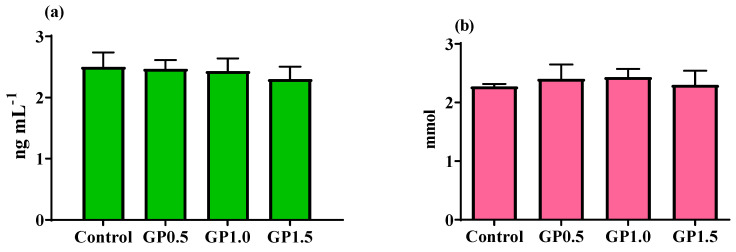
Concentrations of malondialdehyde (MDA) (**a**) and total antioxidant capacity (TAC) (**b**) in Japanese quails fed different levels of GP. Values (means ± SEM) within the same week with uncommon letters are significantly (*p* < 0.05) different.

The effect of experimental diets on blood parameters of animals are presented in Table 6 and Figure 1. The results revealed that supplementation of ginger powder in the diet had significant effect on the level of triacylglycerol (*p* < 0.05). Nevertheless, serum albumin, cholesterol, glucose, MDA, and TAC were not affected by the diet (*p* > 0.05). Level of serum TAC only numerically elevated, while MDA level was reduced in birds fed medium level of ginger powder compared with the control group. The effect of experimental treatments on phenolic compounds of the yolk, as shown in Figure 1, was significant (*p* < 0.05). Highest concentration of ginger powder (1.5 g/kg diet) was the most effective treatment in stimulating the production of phenolic.

## 4. Discussion

In general, inclusion of ginger powder in the diet of birds was effective on various productive parameters. In this experiment, production rate and egg mass were not influenced by the treatments, while feed intake and FCR decreased when birds received increasing levels of ginger powder. In agreement to our results, previous findings confirmed that dietary supplementation with ginger root improved FCR in broiler chickens [39] and decreased feed intake in guinea fowl [40]. Furthermore, ginger supplements had no adverse effect on the palatability of broiler feeds [39] and improved the digestibility of dry matter in guinea fowl [40]. Contrarily, Damaziak et al. [29] did not observe any positive effect of the ginger root on egg production rate in laying hens. However, findings of our study were not in agreement with the handful studies showing the insignificant effect of ginger powder [41] or ginger extract dietary supplementation [42] on the FCR. Akbarian et al. reported that using different levels of ginger (0.25, 0.5 and 0.75 g/100 g of diet) in laying hens during 30 weeks had insignificant effect on FI and FCR [43]. The disparity of this study with previous research results could be due to differences in ginger source, processing methods as well as poultry species [42,44]. Ginger contains many active compounds (e.g., brunel, camphon, limonene, humolin, gingerol, gingeron, gingerdiol, shogaols, some phenolic ketone derivatives, volatile oils, alkaloids, saponins, and flavonoids) [45] that could stimulate feed digestion and the digestive enzymes, thus increasing FI and FCR [38]. In the same way Platel and Srinivasan [46] stated that ginger enhanced the activity of pancreatic lipase, amylase, trypsin chymotrypsin, and bile acid secretion in albino rats. In fact, these enzymes, bile and biliary bile acids significantly affect the digestion and absorption of nutrients. In accord, Habibi et al. [28] indicated the stimulating effect of ginger root powder (7.5 g/kg of diet) on body weight and weight gain in broiler chicks at 22 days of the experiment. In another study, ginger powder increased production, hatchability, reproductive performance, and economic efficiency at a level of 0.05 g/kg diet while increasing egg weight and feed intake in Japanese quail [25]. In present study, ginger powder supplemented diet had no effect on egg production, egg weight, and total egg mass among the treatments during the experiment period. Our findings were in line with the results of Wen at al. [42] and Herve et al. [47] reporting the insignificant effect of ginger supplemented diet in laying hen and quail. Moreover, Wen et al. concluded that egg weight was improved in ginger powder fed laying hen [47]. The results of studies on the effect of ginger at increasing levels of 0, 5, 10, 15, and 20 g/kg of diet on the performance of laying hens at the age of 27 weeks showed that all laying hens were in good health and no mortality was recorded in the whole period of the experiment. Average egg weight and laying rate were similar in treatments containing ginger powder. Egg mass was positively affected by the treatments compared with the control group, which can be related to the positive effect of ginger powder on laying rate as well as egg weight [48]. In contrast to the current quail study, egg production rate was increased in ginger extract/powder fed laying hen [48,49] alone or in combination with medicinal herbs [27]. This means that the beneficial effect of ginger on performance depends on the bird species, dosage of ginger, and its derivatives and interaction with other dietary components. However, information about mechanism of action of ginger intake are scarce [50].

In this study ginger powder was beneficial on the reducing the blood triacylglyceride levels yet its effects were not significant on serum cholesterol and glucose levels. Other serum parameters including albumin, and protein were not significantly affected by ginger powder (*p* > 0.05). Consistent with these results, studies have shown that diet supplementation with ginger extract at 0.4 and 0.6 mg/g of diet, significantly decreased glucose, triglyceride, and cholesterol levels [51]. Akhany et al. [52] concluded that ginger extract significantly reduced blood glucose levels and increased insulin levels. Some of the essential minerals (calcium, zinc, potassium, manganese, and chromium) are related to the mechanism of insulin release [53]. In another study, ginger at concentrations up to 2% of the diet reduced cholesterol, triglyceride, and glucose levels in comparison with the control group, while the serum protein was not affected by the experimental treatments [38]. Ginger has previously shown strong anti-lipidemic effect on serum cholesterol and triglyceride levels [54]; hence, its mode of action may be related to the inhibition of cholesterol synthesis (e.g., β-hydroxy-β-methylglutaryl coenzyme A (HMG—CoA) [51]. Correspondingly, ginger is a potent HMGR-inhibiting drug, known to cause liver-specific inhibition of cholesterol synthesis [55]. In addition, other reports showed that diabetic therapy with insulin helps in reducing the plasma triglycerides by affecting lipoprotein lipase levels [56]. Ginger has insulin-stimulating effect which could reduce plasma triglycerides [56]. Previous studies stated that phenols and flavonoids act as potent antioxidants [57]. Khalifa and Noseer [58] revealed that eggs produced by quail supplemented with combined ginger powder and probiotics had the lowest total cholesterol content in serum and yolk compared with the control, along with an increase in high-density lipoprotein (HDL) and decrease of low-density lipoprotein (LDL). Herve et al. [59] showed that total cholesterol and triglycerides, transaminases, and MDA decreased in quails supplemented with ginger essential oil at 50, 100, and 150 μL/kg body weight.

Haugh unit, albumen height, and yolk index are characterized as main reference associated with egg quality, which influenced by feed additive (e.g., green tea powder) [60]. In this study yolk index, albumen height, and Haugh unit of eggs were increased in diet supplemented with ginger powder. Several studies indicated that ginger supplementation in poultry diets significantly increased antioxidant enzymes as well as TAC and decreased MDA [22,28,43]. Improvement of egg yolk index and Haugh unit in the current study may be due to the effect of the phenolic compounds of ginger (gingerols and shagaol) which have antioxidant properties [61]. As discussed earlier, color of the yolk an acceptability and freshness feature of the egg, could be improved by natural products such as carotenoids. In this study, egg yolk color was positively affected by ginger powder consumption (1.5 mg/kg). However, others have reported the ineffectiveness of fermented ginger powder (10 and 50 mg/kg of diet concentrations) [23] and ginger extract [42] on the yolk color and yolk and albumen percentage. The intensity of egg yolk color depends on the presence and utilization of pigments in the diet, because laying hens have no ability to produce pigments through their biochemical processes [62]. The darker yolk color of eggs from quails received ginger is probably due to the natural pigments found in ginger, including 6-dehydrogingerdione, which causes a deep yellow color [63]. No difference in other quality traits of quail eggs indicates that ginger does not affect the egg shell quality or egg composition (percentage of albumen, yolk, and shell). The results of present experiment consistent with the result of Wen et al. 2019 [42] showing that the use of ginger root as an additive in Japanese quail diet had significant effect on Haugh unit and albumen height yet being ineffective on shell thickness, shell strength, and egg composition percentage. We, in this study, observed concentration dependent potency of ginger powder on many important parameters of fresh egg quality (e.g., Haugh unit, yolk index, and yolk color) in laying quails; however, other variables associated with egg shelf life should be tested for a final statement.

## 5. Conclusions

The results of this study suggest that the inclusion of ginger root powder in Japanese quail diet can partially improve the productive performance, antioxidant status, and blood parameters. Moreover, ginger could improve yolk color and albumen quality expressed in Haugh units. The effects on the quail production performance seemed to be dose dependent and ginger at the highest tested level (1.5 g/kg of ginger powder) was most effective treatment. However, further studies are needed to conclude on the effect of ginger on quality of the poultry products as well as its mechanism of action.

## Figures and Tables

**Table 1:** Ingredient and composition of the basal diet.

Item	Value (%)	Diet Composition	Value
Corn	45.38	ME (kcal/kg)	2900
Soybean meal	32.50	Crude protein (%)	20
Wheat bran	10.51	Ca (%)	2.5
Soybean oil	3.2	Available P (%)	0.39
Di calcium phosphate	1.8	Na	0.15
Calcium carbonate	5.3	Methionine (%)	0.7
Methionine	0.39	Lysine (%)	1.12
Sodium chloride	0.32	Sys (%)	0.34
Bicarbonate	0.1		
Vitamin ^1^	0.3		
Trace mineral ^2^	0.3		

^1^ Supplied per kg of feed: vitamin B1, 2.16 mg; vitamin B2, 7.92 mg; pantothenic acid, 12 mg; nicotinic acid;36, vitamin B6, 3.6 mg; folic acid, 1.2 mg; biotin, 0.12 mg; vitamin K3, 2.4 mg; vitamin E, 21.6 IU; chloin chloride, 300; antioxidant, 120 mg; vitamin A, 10,800 IU; vitamin D3, 2400 IU and vitamin B12, 0.018 mg. ^2^ Supplied per kg feed: FeSO_4_, 0.15 g; MnSO_4_, 0.12 g; CuSO_4_, 0.03 g; I, 1.2 mg and Se, 0.24 mg.

**Table 2:** Nutrient composition of ginger powder (% DM) used in the experiment.

Item	Level (%)
Dry matter, DM	88.5
Phenol, component (g/100 gr)	0.703
Crude protein	5.5
Ash	11.75
Organic matter, OM	88.25
Ether extract, EE	1.35
Neutral detergent fiber, NDF	7.5
Acid detergent fiber, ADF	4.5
Natural detergent soluble fiber, NDS	92.5
Acid Detergent soluble fiber, ADS	95.5

**Table 3:** Effects of ginger powder supplementation on productive performance in Japanese quails.

Item		Feed Intake (g/day)	Feed Conversion Ratio (g:g)	Egg Production Rate (%)	Egg Weight (g)	Egg Mass (%)
Treat						
Control		33.87 ^a^	3.03	92.99	12.01	11.16
GP0.5		32.93 ^b^	2.88	94.10	12.13	11.41
GP1.0		32.19 ^c^	2.77	94.42	12.31	11.62
GP1.5		31.33 ^d^	2.72	94.77	12.15	11.51
SEM		0.17	0.02	1.19	0.13	0.13
Week 0		34.02	2.99	93.87	12.12	11.37
Time, w						
Week 2		34.21 ^a^	2.95	94.51	12.25 ^a^	11.58 ^a^
Week 4		32.13 ^b^	2.78	94.91	12.17 ^a^	11.54 ^a^
Week 6		32.06 ^b^	2.85	93.84	11.99 ^b^	11.25 ^b^
Week 8		31.93 ^b^	2.82	93.03	12.18 ^a^	11.33 ^b^
SEM		0.21	0.01	0.82	0.08	0.08
treat × Time						
Control	Week 2	34.71	3.02 ^a,b^	93.92	12.22	11.48
Control	Week 4	34.45	3.01 ^a,b,c^	94.10	12.13	11.41
Control	Week 6	33.66	3.1 ^a^	92.32	11.77	10.85
Control	Week 8	32.68	2.99 ^a,b,c,d^	91.61	11.91	10.91
GP0.5	Week 2	34.82	3.05 ^a,b^	93.21	12.26	11.42
GP0.5	Week 4	32.11	2.81 ^d,e,f^	94.46	12.08	11.39
GP0.5	Week 6	31.94	2.85 ^b,c,d,e,f^	93.03	12.02	11.18
GP0.5	Week 8	32.84	2.82 ^c,d,e,f^	95.71	12.15	11.63
GP1.0	Week 2	34.05	2.89 ^b,c,d,e^	95.17	12.36	11.77
GP1.0	Week 4	31.19	2.69 ^f,g^	94.28	12.26	11.56
GP1.0	Week 6	32.03	2.77 ^e,f,g^	94.50	12.23	11.56
GP1.0	Week 8	31.50	2.71 ^e,f,g^	93.75	12.37	11.59
GP1.5	Week 2	33.25	2.85 ^b,c,d,e,f^	95.71	12.15	11.65
GP1.5	Week 4	30.80	2.61 ^g^	96.78	12.19	11.80
GP1.5	Week 6	30.58	2.68 ^f,g^	95.53	11.94	11.42
GP1.5	Week 8	30.69	2.74 ^e,f,g^	91.07	12.31	11.19
SEM		0.42	0.03	1.64	0.16	0.17
Probability						
Treat		>0.0001	>0.0001	0.74	0.48	0.16
Time		>0.0001	>0.0001	0.32	0.001	0.007
Treat × Time		0.07	0.01	0.23	0.41	0.06

Different letters (^a, b, c, d, e, f^ or ^g^) after the means within a column indicate significant differences among treatments (*p* < 0.05).

**Table 4:** Effect of different level of ginger root powder on egg quality of Japanese quails.

Traits	Experimental Diets					*p* Value		
	Ginger Powder (g/kg of Diet)				SEM	Treatment	Time	Treatment × Time
	0	0.5	1	1.5				
Shape index %	128.97	130.25	253.90	122.66	62.09	0.43	0.45	0.43
Albumen index %	10.36	10.74	10.89	10.79	0.23	0.46	0.01	0.009
Yolk index %	44.77 ^c^	47.02 ^a^	46.16 ^a,b^	45.17 ^b,c^	0.54	0.005	>0.001	0.13
Albumen height (mm)	3.81 ^b^	3.99 ^a^	4.11 ^a^	4.04 ^a^	0.04	0.004	0.21	0.02
Haugh unit	84.88 ^b^	86.06 ^a^	86.62 ^a^	86.27 ^a^	0.24	0.003	0.13	0.07
Albumen weight	7.45	7.49	7.52	7.53	0.09	0.93	0.49	0.15
Yolk colour	4.25 ^b^	4.45 ^b^	4.70 ^a^	4.75 ^a^	0.08	0.004	>0.001	>0.001
Shell weight (g)	0.965	0.966	0.987	0.989	0.01	0.56	0.59	0.98
Albumen percentage %	59.92	60.80	60.14	60.41	0.36	0.39	0.11	0.29
Yolk percentage %	32.31	31.35	31.95	31.64	0.30	0.20	0.03	0.44
Shell percentage %	7.76	7.84	7.90	7.93	0.11	0.73	0.59	0.29
Albumen to yolk	0.539	0.515	0.531	0.524	0.008	0.26	0.03	0.33
Yolk weight (g)	4.02	3.86	3.99	3.94	0.05	0.21	0.08	0.70
Yolk pH	5.87 ^a^	5.84 ^b^	5.81 ^b^	5.85 ^b^	0.01	0.02	>0.001	0.02
Albumen pH	8.91 ^a^	8.78 ^b^	8.77 ^b^	8.83 ^b^	0.02	0.01	>0.001	0.009
Egg specific gravity (g/cm^3^)	1.0740	1.0745	1.0748	1.0750	0.0006	0.72	0.60	0.97
Shell thickness (mm × 10)^2^	20.61	20.67	20.29	20.76	0.35	0.80	0.005	0.51
Shell strength (hg/cm^2^)	19.51	19.71	19.86	19.94	0.28	0.73	0.59	0.97

Different letters (^a, b^ or ^c^) after the means within a row indicate significant differences among treatments (*p* < 0.05).

**Table 5:** Effect of treatment and time Interaction on egg albumen and yolk traits in Japanese quails fed different level of ginger root powder.

Treat	Time (Week)	Albumen Index	Albumen Height (mm)	Yolk Colour	Yolk pH	Albumen pH
Control	2	10.38 ^a,b^	3.71 ^b^	3.87 ^d^	5.91 ^a,b^	8.99 ^a,b^
Control	4	9.75 ^b^	3.68 ^b^	4.56 ^b,c^	5.85 ^a,b,c,d^	9.10 ^a^
Control	6	10.36 ^a,b^	3.90 ^a,b^	4.35 ^b,c,d^	5.84 ^a,b,c,d^	8.81 ^c,d,e,f^
Control	8	10.95 ^a,b^	3.94 ^a,b^	4.20 ^b,c,d^	5.89 ^a,b,c^	8.75 ^c,d,e,f^
GP0.5	2	9.98 ^a,b^	4.01 ^a,b^	4.20 ^b,c,d^	5.81 ^b,c,d^	8.73 ^e,f,g^
GP0.5	4	11.09 ^a,b^	4.001 ^a,b^	4.12 ^c,d^	5.82 ^a,b,c,d^	8.91 ^a,b,c,d^
GP0.5	6	10.86 ^a,b^	3.91 ^a,b^	4.56 ^b,c,d^	5.85 ^a,b,c,d^	8.74 ^e,f,g^
GP0.5	8	11.02 ^a,b^	4.04 ^a,b^	4.91 ^a,b^	5.88 ^a,b,c^	8.72 ^e,f,g^
GP1.0	2	10.99 ^a,b^	4.26 ^a^	4.29 ^b,c,d^	5.76 ^d^	8.76 ^c,d,e,f^
GP1.0	4	10.33 ^a,b^	3.98 ^a,b^	4.41 ^b,c,d^	5.81 ^a,b,c,d^	8.90 ^a,b,c,e^
GP1.0	6	11.50 ^a^	4.25 ^a^	4.54 ^b,c,d^	5.81 ^a,b,c,d^	8.72 ^d,f,g^
GP1.0	8	10.71 ^a,b^	3.94 ^a,b^	5.56 ^a^	5.86 ^a,b,c,d^	8.70 ^f^
GP1.5	2	10.24 ^a,b^	4.05 ^a,b^	4.35 ^b,c,d^	5.85 ^a,b,c,d^	8.81 ^b,c,d,e,f^
GP1.5	4	10.45 ^a,b^	4.04 ^a,b^	4.50 ^b,c,d^	5.79 ^c,d^	8.89 ^a,b,c,d,e,f^
GP1.5	6	11.26 ^a,b^	4.06 ^a,b^	4.58 ^b,c,d^	5.84 ^a,b,c,d^	8.76 ^c,d,e,f^
GP1.5	8	10.87 ^a,b^	4.02 ^a,b^	5.56 ^a^	5.92 ^a^	8.86 ^b,c,d,e,f^
SEM		0.34	0.08	0.13	0.02	0.04
*p* value		0.009	0.02	0.001	0.02	0.009

Different letters (^a, b, c, d, e, f^ or ^g^) after the means within a column indicate significant differences among treatments (*p* < 0.05).

**Table 6:** Biochemical parameters in blood serum of Japanese quail fed diets with ginger powder supplementation.

Treatments	Protein (g dL^−1^)	Albumin (g dL^−1^)	Triacylglycerol (mg dL^−1^)	Cholesterol (mg dL^−1^)	Glucose (mg dL^−1^)
Control	5.80	1.60	453.1 ^a^	243	195
Ginger (0.5 g/kg of diet)	5.70	1.43	351.8 ^b^	247	242
Ginger (1 g/kg of diet)	5.73	1.56	336.8 ^b^	210	173
Ginger (1.5 g/kg of diet)	6.15	1.70	342.6 ^b^	219	221
SEM	0.28	0.1	23.03	22.18	21.80
*p*-value	0.64	0.39	0.01	0.58	0.17

Different letters (^a^ or ^b^) after the means within a column indicate significant differences among treatments (*p* < 0.05).

## Data Availability

Qualified researchers can obtain the data from the corresponding author. The data are not publicly available due to privacy concerns imposed by the RBCT ethical principles.
